# Hematological factors associated with immunity, inflammation, and metabolism in patients with systemic lupus erythematosus: Data from a Zhuang cohort in Southwest China

**DOI:** 10.1002/jcla.23211

**Published:** 2020-01-24

**Authors:** Xiaoxia Lao, Liping Ma, Qingwei Ma, Qiaorong Ma, Zhige Yang, Lingxiao Guo, Wenzheng Nong

**Affiliations:** ^1^ Department of Clinical Laboratory Minzu Hospital of Guangxi Zhuang Autonomous Region Affiliated Minzu Hospital of Guangxi Medical University Guangxi China; ^2^ Department of Gynaecology Minzu Hospital of Guangxi Zhuang Autonomous Region Affiliated Minzu Hospital of Guangxi Medical University Guangxi China

**Keywords:** association rule, hematological parameters, immunity, inflammation, metabolism, systemic lupus erythematosus

## Abstract

**Introduction:**

Hematological parameters play important role in multiple diseases. This study was to investigate the possible association of the routine hematological parameters involved in immunity, inflammation, and metabolism with systemic lupus erythematosus (SLE) in patients of Zhuang ethnicity in Guangxi, southwest China.

**Methods:**

The medical records of 195 Zhuang SLE patients between January 2013 and November 2018 were retrospectively reviewed. Random forest algorithm and multivariate logistic regression were used to identify the feature hematological parameters in patients with SLE. Association rules were explored between each parameter and immunity‐ (IgG, IgA, IgM, C3, and C4), inflammation‐ (ESR, hs‐CRP, and CAR), and metabolism‐ (TG, TC, HDL‐C, LDL‐C, TP, PA, ALB, and UA) related indexes.

**Results:**

Random forest algorithm and logistic regression analysis showed that neutrophil‐to‐lymphocyte ratio (NLR), red blood cell distribution width (RDW), and platelet‐to‐lymphocyte ratio (PLR) were the feature parameters for distinguishing SLE patients from healthy controls. According to the ROC curves, the optimal cutoff values to predict SLE were 1.98 for NLR, 13.35 for RDW, and 145.64 for PLR. Association rule analysis showed that NLR was strongly associated with C3, hs‐CRP, TG, ALB, and UA; RDW was strongly associated with C3, C4, hs‐CRP, TG, and ALB; PLR was strongly associated with IgG, hs‐CRP, HDL‐C, and UA.

**Conclusions:**

Neutrophil‐to‐lymphocyte ratio, RDW, and PLR may serve as effective predictors of dysregulation in immunity, inflammation, and metabolism. These three indicators may be potential for cardiovascular risk assessment in Zhuang SLE patients in southwest China.

## 1 INTRODUCTION

Systemic lupus erythematosus (SLE) is a chronic inflammatory autoimmune disease affecting different organs and has various clinical manifestations. Hematological manifestation is confirmed as the most common initial presentation in SLE, including anemia, thrombocytopenia, leukopenia, and lymphopenia.^1^ Nearly all SLE patients develop hematological abnormalities during their disease course, either isolated or in conjunction with other manifestations.^2^

The cardiovascular involvement in SLE and the subsequent cardiovascular disease predispose to a significant morbidity and can raise the mortality risk, which occurs more often late in active SLE states.^3^ The proportion of cardiovascular events is higher in SLE than in general populations of comparable age and sex.^4^ Abnormal immune activation, chronic inflammatory state, endothelial dysfunction, and metabolic disorders have been proven to raise the risk of cardiovascular events in SLE.^5^, ^6^

Many different markers such as immunoglobulin, complement, c‐reactive protein, erythrocyte sedimentation rate, interleukin, and interferon have been used to assess immune and inflammatory status in multiple diseases. Recently, hematological parameters have received more attention, which include red blood cell distribution width (RDW), neutrophil‐to‐lymphocyte ratio (NLR), platelet‐to‐lymphocyte ratio (PLR), mean platelet volume (MPV), hematocrit (HCT), and eosinophil‐to‐lymphocyte ratio (ELR). These parameters have been novel biomarkers in diagnosis, prognosis, risk stratification, and predicting survival and mortality in a variety of diseases, such as cardiovascular diseases,^7^ cancers,^8^ autoimmune diseases,^9^ and parasitic diseases.^10^ In recent years, RDW, NLR, and PLR have played an active role in inflammations and had regulatory effects on the immune system in autoimmune diseases. Previous studies have showed that RDW, NLR, and PLR could be used for evaluating inflammatory response, disease activity, and infection in rheumatoid arthritis, dermatomyositis, and SLE patients.^11^, ^12^, ^13^

Nevertheless, the correlation between hematological parameters and cardiovascular involvement in SLE patients has not yet been elucidated. Hence, the aim of the present study was to explore the feature hematological parameters associated with SLE and to investigate the possible association of hematological parameters involved in immune, inflammatory, and metabolic indexes related to cardiovascular risk in a Zhuang SLE cohort.

## 2 MATERIALS AND METHODS

### 2.1 Participants

A total of 195 SLE patients were originally recruited to the Department of Rheumatology at Minzu Hospital of Guangxi Zhuang Autonomous Region between January 2013 and November 2018. All patients were newly diagnosed without treatment based on the SLE diagnostic criteria established by the American College of Rheumatology.^14^ Patients were excluded if they had received a blood transfusion, had any other autoimmune disease, or had malignancy, diabetic nephropathy, lymphoproliferative disorder, hepatosplenic or hematologic disease, or active infection. Additionally, 183 age‐ and sex‐matched healthy subjects who had undergone routine health examination in the same hospital during the same period were enrolled as the control group. All participants were from the Zhuang ethnic minority in Guangxi, China. The study was approved by the ethics committee of Minzu Hospital of Guangxi Zhuang Autonomous Region.

### 2.2 Data extraction

Demographic characteristics, clinical data, and laboratory findings for each participant were extracted from electronic medical records at Minzu Hospital of Guangxi Zhuang Autonomous Region. Following data were collected: (a) immune indexes: immunoglobulin G (IgG), immunoglobulin A (IgA), immunoglobulin M (IgM), complement 3 (C3), and complement 4 (C4); (b) inflammatory indexes: hypersensitive c‐reactive protein (hs‐CRP), erythrocyte sedimentation rate (ESR), c‐reactive protein (CRP), and proportion of c‐reactive protein level to albumin level (CAR); (c) metabolic indexes: triglyceride (TG), total cholesterol (TC), high‐density lipoprotein cholesterol (HDL‐C), low‐density lipoprotein cholesterol (LDL‐C), uric acid (UA),^15^ total protein (TP), albumin (ALB), and prealbumin (PA); (d) hematological parameters: white blood cell (WBC), neutrophil (NEU), lymphocyte (LYM), red blood cell (RBC), hemoglobin (HGB), hematocrit (HCT), mean corpuscular volume (MCV), red blood cell distribution width (RDW), platelet (PLT), mean platelet volume (MPV), platelet distribution width (PDW), plateletcrit (PCT). NLR was calculated as the proportion of absolute neutrophil count to lymphocyte count. PLR was calculated as the proportion of platelet count to lymphocyte count. Additionally, SLE disease activity was evaluated by SLE Disease Activity Index (SLEDAI) score.^16^

### 2.3 Data analysis

#### 2.3.1 Random forest algorithm

Random forest is an ensemble classifier used for data mining, it is composed of numerous decision trees, each one relying on the values of a random vector sampled independently. Based on the random forest algorithm, the out‐of‐bag (OOB) classification error rate was calculated. Subsequently, Mean Decrease Gini (MDG), the total decrease in node impurities measured by the Gini index from splitting on variables and averaged over all trees, was calculated.^17^ It provides a way to quantify which parameters contribute most to the accuracy of classification, and greater MDG will suggest an important feature parameter. In this way, we used MDG to rank the important feature hematological parameters with SLE patients. Statistical analysis was carried out using randomForest package of R software (http://www.r-project.org).

#### 2.3.2 Association rules analysis

In order to systematically verify the associations between hematological parameters and immunity, inflammation, and metabolism indexes, association rules analysis was used to estimate the strength of the associations. The Apriori algorithm is often used to discover association rules.^18^ For association rules models, the immune, inflammatory, and metabolic indexes and the feature hematological parameters are set as former and consequent item, respectively. An association rule reflects the interdependence and correlation between two variables. The strength of an association rule in the Apriori algorithm is determined by its support and confidence. By setting the minimum support and confidence thresholds, we aim to mine more meaningful association rules. We performed this statistical analysis with the arules package of R software (http://www.r-project.org).

#### 2.3.3 Statistical analysis

The student's *t* test or the Mann‐Whitney *U* test was performed to compare differences between the two groups based on distribution status. Further, Spearman's correlation coefficient was used to evaluate the correlations between two variables. A multivariate logistic regression was performed to determine which hematologic parameters were best associated with SLE, and ROC curves were created to analyze optimal cutoff value, sensitivity, and specificity of the parameters in predicting SLE *P* < .05 was regarded as statistically significant, and all statistical analysis was conducted using SPSS (version 17.0, SPSS Inc).

## 3 RESULTS

### 3.1 Characteristics of the subjects

**Figure 1:**
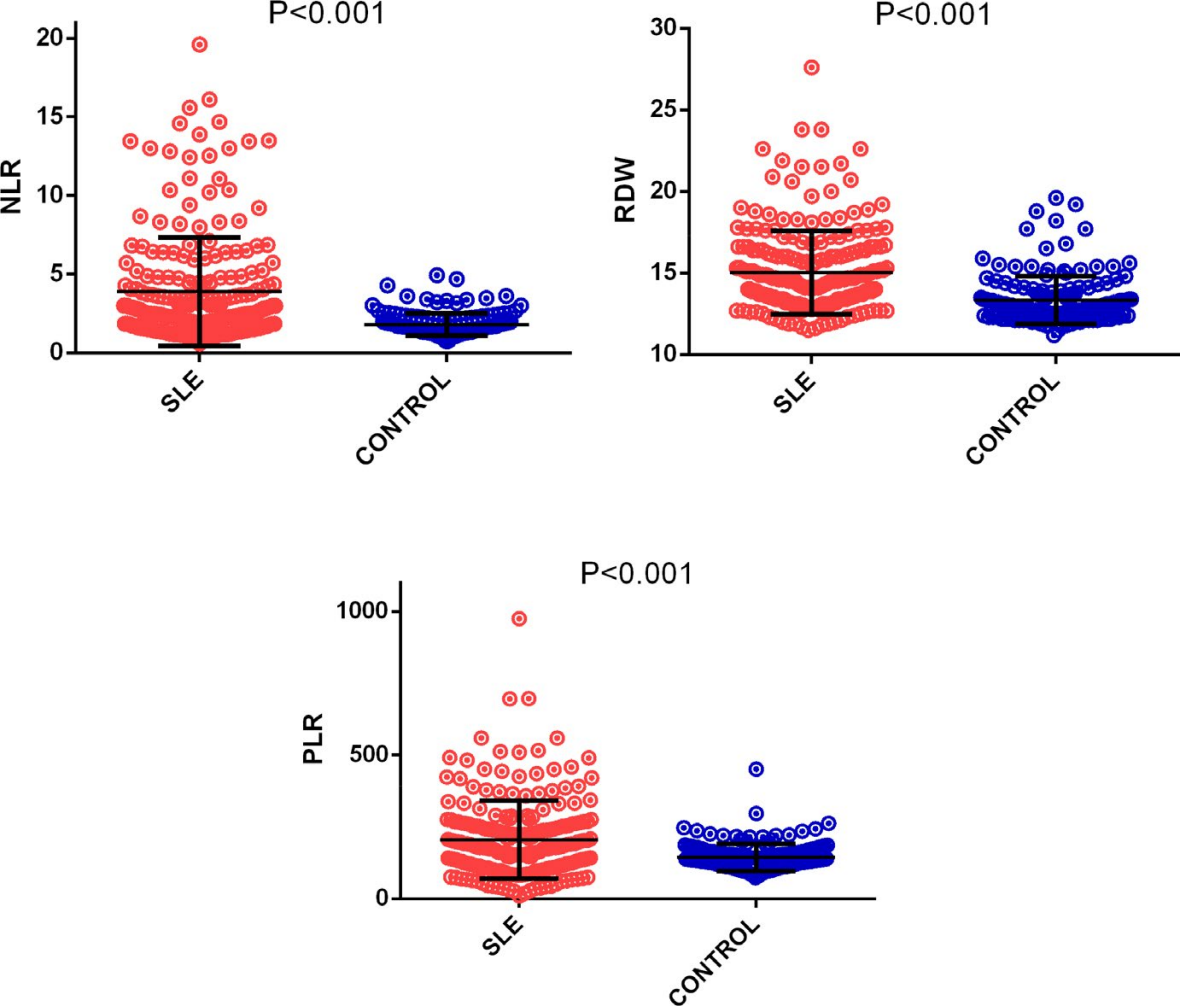
Comparison of NLR (neutrophils‐to‐lymphocytes ratio), RDW (red blood cell distribution width), and PLR (platelet‐to‐lymphocyte ratio) levels in SLE patients and healthy controls

The demographic and clinical characteristics and the laboratory data of the study population are summarized in Table S1. In the patient group, WBC, neutrophils, lymphocytes, RBC, HGB, HCT, MCV, and PCT levels were significantly decreased compared with those in the control group, while RDW, NLR, and PLR levels were significantly higher (Figure 1). In addition, hs‐CRP, ESR, CAR, IgG, TC, TG, and UA levels were significantly higher and TP, PA, ALB, C3, C4, and HDL‐C levels were significantly lower in the SLE group as compared to the controls.

### 3.2 Hematological parameters for characterizing SLE patients

#### 3.2.1 Random forest algorithm

**Figure 2:**
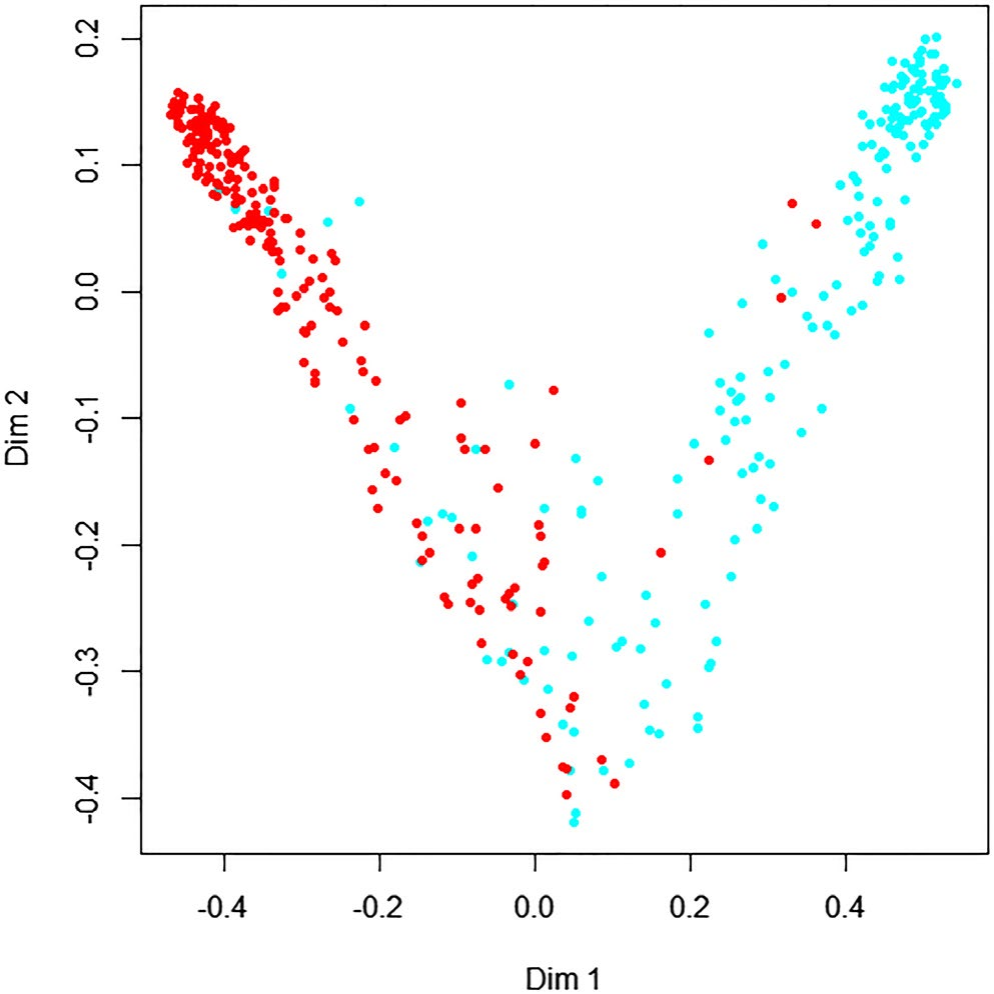
Multi‐dimensional scaling graph of the hematological parameters. The abscissa and longitudinal coordinates indicate two dimensionalities; the red dogs and blue dots indicate SLE and healthy controls, respectively

We applied the random forest algorithm by constructing 5000 decision trees from which a relatively stable OOB classification error rate of 7.33% could be obtained. The multi‐dimensional scaling (MDS) plot of the proximity matrix for the hematological parameters was depicted by this random forest, showing similarities among samples and their respective categories by projecting a high‐dimensional measure to a two‐dimensional surface. This graph displayed good classification effects between SLE patients and healthy controls (Figure 2).

**Figure 3:**
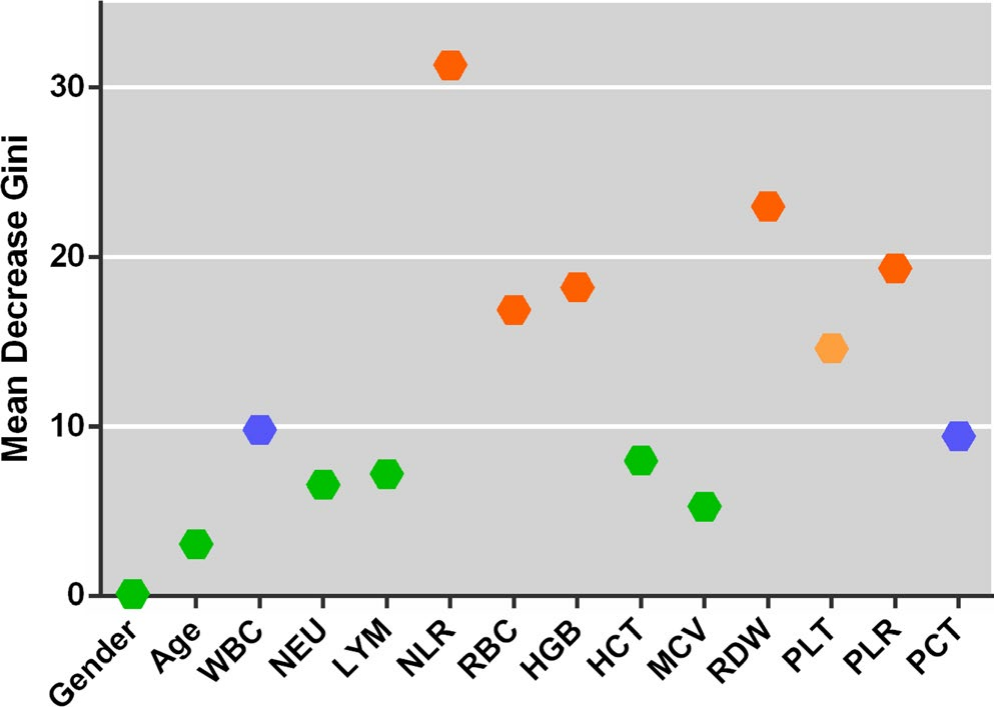
Comparison of Mean Decrease Gini values for hematological parameters in systemic lupus erythematosus patients

Based on MDG analysis, we found that NLR, RBC, RDW, HGB, and PLR had larger MDG values than the other hematological parameters (Table 1). This suggested that these five parameters were the most important hematological characteristics associated with SLE patients (Figure 3).

#### 3.2.2 Multivariate logistic regression

The statistically significant hematological parameters shown in Table S1 were selected for multivariate logistic regression analysis. The results were presented in Table 2, which showed NEU (Exp(B) = 0.217, *P* = .008), NLR (Exp(B) = 4.028, *P* = .001), RBC (Exp(B) = 0.041, *P* = .000), RDW (Exp(B) = 2.008, *P* = .000), PLT (Exp(B)=0.971, *P* = .000), and PLR (Exp(B) = 1.021, *P* = .000). These results revealed that increased NLR, RDW, and PLR were significantly correlated with the occurrence of SLE.

Hence, by means of random forest algorithm in conjunction with multivariate logistic regression analysis, the results demonstrated that increased NLR, RDW, and PLR were the important feature parameters associated with SLE patients.

### 3.3 AUC, sensitivity, and specificity

**Figure 4:**
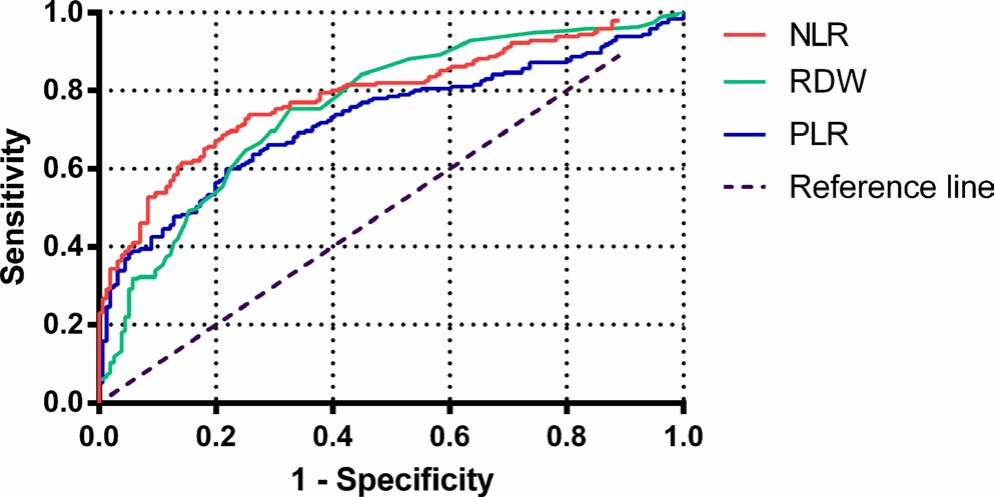
ROC curves of neutrophil‐to‐lymphocyte ratio, red blood cell distribution width, and platelet‐to‐lymphocyte ratio for differentiating systemic lupus erythematosus patients

ROC curves were developed by comparing the NLR, RDW, and PLR results of SLE patients with those of healthy controls (Figure 4). The optimal cutoff values for these three parameters were determined by the maximum Youden index accumulated by the ROC curves. Our results showed that the optimal thresholds for NLR, RDW, and PLR were 1.98, 13.35, and 145.64, respectively. For NLR, the AUC value was 0.79 with 75.8% sensitivity and 74.4% specificity. RDW produced an AUC value of 0.76 with sensitivity and specificity of 75.4% and 69.3%, respectively. The AUC value for PLR was 0.72 with 73.8% sensitivity and 68.4% specificity.

### 3.4 Correlation of NLR, RDW, and PLR with immune, inflammatory, and metabolic indexes

As shown in Table 3, NLR demonstrated a significant positive correlation with ESR (*r* = .635, *P* = .000), hs‐CRP (*r* = .469, *P* = .000), CAR (*r* = .244, *P* = .003), TG (*r* = 383, *P* = .000), UA (*r* = .374, *P* = .000), and SLEDAI (*r* = .346, *P* = .000), whereas it displayed a negative correlation with C3 (*r* = −0.265, *P* = .000), LDL (*r* = −.237, *P* = .000), and ALB (*r* = −.364, *P* = .000). RDW showed a positive association with hs‐CRP (*r* = .259, *P* = .017), CAR (*r* = .248, *P* = .001), TG (*r* = .326, *P* = .000), and SLEDAI (*r* = .279, *P* = .000), while a negative association with C3 (*r* = −.207, *P* = .002), C4(*r* = −.196, *P* = .004), TP (*r* = −.257, *P* = .001), and ALB (*r *= −.192, *P* = .006). PLR was positively correlated with IgG (*r* = .263, *P* = .000), hs‐CRP (*r* = .193, *P* = .012), and SLEDAI (*r* = .312, *P* = .000), but negatively correlated with HDL (*r* = −.275, *P* = .000), PA (*r* = −.275, *P* = .041), and UA (*r* = −.187, *P* = .008).

### 3.5 Association rules between NLR, RDW, and PLR and immune, inflammatory, and metabolic indexes

For association rules models, the immune, inflammatory, and metabolic indexes and the feature hematological parameters were set as former and consequent item, respectively. The strength of an association rule was determined by its support and confidence. In this experiment, the minimum support and confidence degree were set at 20% and 50%, respectively. After executing the association model, 14 association rules were searched out (Table 4). The results showed that all support levels were >20% and confidence levels were >50%, which indicated that NLR was strongly associated with C3, hs‐CRP, TG, ALB, and UA; RDW was strongly associated with C3, C4, hs‐CRP, TG, and ALB; and PLR was strongly associated with IgG, hs‐CRP, HDL‐C, and UA.

## 4 DISCUSSION

Cardiovascular events are proportionally higher in SLE patients compared with general populations of comparable age and sex.^4^ Abnormal immune activation, chronic inflammatory state, and metabolic disorders have been proven to raise the risk of cardiovascular events in SLE.^5^, ^6^ The aim of the current study was to investigate the association of hematological parameters involved in immune, inflammatory, and metabolic indexes related to cardiovascular risk in a Zhuang SLE cohort. As an important finding, this study shows that increased NLR, RDW, and PLR are the important feature hematological parameters for distinguishing SLE patients from healthy controls. Another novel finding is that increased NLR, RDW, and PLR are significantly associated with immune, inflammatory, and metabolic dysregulation. These results imply that increased NLR, RDW, and PLR might be potential indexes for assessing cardiovascular risk in Zhuang SLE patients.

The diagnostic value of NLR, RDW, and PLR has been explored in this study. Based on the ROC curve, we found that the AUC value for NLR was 0.79 with 75.8% sensitivity and 74.4% specificity. RDW produced an AUC value of 0.76 with 75.4% sensitivity and 69.3% specificity. The AUC value for PLR was 0.72 with 73.8% sensitivity and 68.4% specificity. Our study demonstrates good sensitivity and specificity as shown by others.^19^ After random forest algorithm and multivariate logistic regression analysis, we found that increased NLR, RDW, and PLR are the important feature parameters associated with SLE patients. These suggest that NLR, RDW, and PLR might serve as valuable complementary biomarkers for SLE.

Hematological abnormalities are very frequently involved in SLE. Anemia is particularly common, followed by leukopenia and thrombocytopenia.^20^ The mechanisms of hematological change in SLE are not fully understood, though some believe that it may be due to immune‐mediated bone marrow depression, excessive peripheral cell destruction, drug damage, stress, or secondary infection.^21^, ^22^ Additionally, polyclonal B‐cell activation is involved in autoimmunity, resulting in the production of cytokines and immunoglobulins. Neutrophils and platelets participate in the production of these cytokines, which, in turn, may be conducive to the further activation of the neutrophils and platelets.^23^

WBC and its subtypes have been used to assess inflammatory and infectious status. NLR, as the ratio of neutrophils over lymphocytes, reflects the balance between innate immunity and adaptive immunity. Although the mechanism is not clear, chronic inflammation appears to influence the relationship between NLR and various outcomes.^24^, ^25^ Recently, a further specific mechanism has been elucidated that NLR level is closely related to the phenotypic activity of granulocytic myeloid‐derived suppressor cells (MDSCs).^26^, ^27^ Common myeloid progenitor cells are more likely to differentiate into MDSCs under chronic pathologic states such as inflammation,^28^ cancer,^29^ and cardiovascular disease.^30^ An up‐regulation in peripheral leukocytes by MDSCs may result in higher neutrophil levels, while lower lymphocyte levels may be due to their suppression by MDSCs, which shows a similar trend to that we observed for NLR values following SLE patients in our study.

An elevated NLR is associated with an increased degree of immune dysfunction and has been identified as a useful predictor of prognosis, survival, and mortality in several conditions including malignancy,^31^ cardiovascular,^23^ and renal diseases.^32^ NLR has also been studied to evaluate inflammatory response and disease activity in autoimmune diseases such as rheumatoid arthritis,^11^ dermatomyositis,^12^ and SLE. A study by Soliman et al^33^ showed that NLR was much higher in SLE patients with active disease and with nephritis; NLR was positively correlated with ESR, CRP, and SLEDAI but negatively correlated with C4. Elsewhere, Qin et al^19^ determined that an NLR level of 2.065 was a predictive cutoff value of SLE with 74.7% sensitivity and 77.5% specificity. Our results are in accordance with these findings, further demonstrating that NLR could be used to reflect immune and inflammatory response in SLE.

Platelets and lymphocytes are thought to be important factors in disease immunology and inflammation. Activated platelets not only produce growth factors but also release chemokines which play significant roles in inflammatory progress. On the other hand, lymphocytes are involved in immune surveillance and immunoediting, and decreased lymphocyte count and function can indicate impairment of immune surveillance and defense.^34^ An increased PLR correlates with poor overall and progression‐free survival in some malignancies such as lung cancer^30^ and cholangiocarcinoma.^35^ PLR has also been validated as an independent risk factor for brain metastasis of lung adenocarcinoma.^36^ Relatively few studies have demonstrated correlations between PLR and autoimmune diseases. Sargin et al^37^ showed that PLR is significantly correlated with results on the disease activity score of 28 joints before and after treatment in patients with rheumatoid arthritis. Soliman et al^32^ reported that PLR is higher in SLE patients, finding it to be positively correlated with ESR, CRP, and SLEDAI, but negatively correlated with C4. In our study, PLR was strongly correlated with hs‐CRP and IgG which confirms that it can be used as a simple biomarker to assess immunity and inflammation in SLE.

Red blood cell distribution width has traditionally served as a useful indicator in diagnosing different types of anemia such as iron deficiency and thalassemia. In addition to carrying oxygen, the most important functions of RBCs are to participate in inflammatory processes and coagulation. A study by Pretorius^38^ found by scanning electron microscopy that the ultrastructure of RBCs changes in inflammatory diseases. An elevated RDW has been found to correlate with pathophysiological states including cardiovascular events,^39^ cancer,^40^ and inflammatory diseases.^41^ Moreover, several studies have shown that an up‐regulated RDW level is significantly associated with inflammation levels in autoimmune diseases. For example, Yujie^41^ found that elevated RDW is correlated with inflammatory markers such as CRP and ESR in patients with rheumatoid arthritis, and Zhide^13^ show that RDW is up‐regulated and positively associated with ESR, CRP, IgM, and SLEDAI in SLE patients. Our findings are similar to previous studies discussed above, suggesting that RDW could be useful in monitoring inflammation and disease progression in SLE patients.

As we know, serum immunoglobulin, complement, c‐reactive protein, erythrocyte sedimentation rate, and SLEDAI scores are often used to assess immune, inflammatory status and evaluate disease activity in SLE patients. In our study, increased IgG, CRP, hs‐CRP, ESR and decreased C3 and C4 levels had been found in SLE patients. After correlation coefficient and association rules analysis, we found that NLR was correlated with ESR, hs‐CRP, CAR, C3, and SLEDAI; RDW was correlated with hs‐CRP, CAR, C3, C4, and SLEDAI; PLR was correlated with hs‐CRP, IgG, and SLEDAI. Theses suggest that NLR, RDW, and PLR are useful reliable markers in the assessment of immune, inflammatory response and disease activity in SLE patients.

Nevertheless, the association of NLR, RDW, and PLR with metabolism in SLE patients has remained unclear. Traditionally, serum triglyceride (TG), total cholesterol (TC), high‐density lipoprotein cholesterol (HDL‐C), low‐density lipoprotein cholesterol (LDL‐C), uric acid (UA),^15^ albumin (ALB), and prealbumin (PA) are often used to evaluate metabolic status. Metabolic disturbance has been strongly correlated with cardiovascular disease, and both have been found to increase with disease duration in SLE. A commonly reported phenotype for Chinese SLE patients is prominent hypertriglyceridemia and low HDL.^42^ In the present study, TC, TG, and UA levels were higher and TP, ALB, PA, and HDL‐C levels significantly lower in the SLE group when compared to the healthy controls. Further correlation coefficient and association rules analysis showed that NLR was correlated with TG, UA, ALB, and LDL. RDW was correlated with TG, TP, and ALB. PLR was correlated with HDL, PA, and UA. The mechanisms through which NLR, RDW, and PLR interacted with the metabolic disturbance were not elucidated. Further research on this is necessary. As such, our study suggests that NLR, PLR, and RDW may be used as novel markers to assess metabolic disorder in SLE patients.

The main advantage of the present study is that NLR, RDW, and PLR can be easily calculated in daily clinical practice and are less costly than other biomarkers such as autoantibodies, immunoglobulin, inflammatory cytokines, and lipid profiles. In addition, these ratios are relatively stable and less affected by physiological, pathological, and physical factors. Moreover, this research has significant clinical implications by providing valuable reference indexes to predict the dysregulation of immunity, inflammation, and metabolism related to the cardiovascular risk in a Zhuang SLE cohort.

However, our study also contains some limitations. First, it is a single‐center study, which limited the ability to reflect the overall situation of SLE patients. Second, some unavoidable inherent defects, such as recall and selection bias, exist in the research. Third, this study was designed as a retrospective examination which lacks longitudinal observation. Therefore, our findings need to be further validated through multi‐center, large population, and prospective studies.

In conclusion, We demonstrated that NLR, RDW, and PLR increased in SLE patients and showed associations with immune, inflammatory, and metabolic indexes. NLR, RDW, and PLR might be effective predictors in immune, inflammatory, and metabolic dysregulation and potential indexes for assessing cardiovascular risk in Zhuang SLE patients in southwest China.

5

**Table 1:** Mean Decrease Gini (MDG) value of hematological parameters in SLE patients

Indexes	gender	age	WBC	NEU	LYM	NLR	RBC	HGB	HCT	MCV	RDW	PLT	PLR	PCT
MDG	0.14	3.07	9.81	6.56	7.21	31.32	16.88	18.20	7.98	5.28	22.98	14.61	19.33	9.43

**Table 2:** Multivariate logistic regression for screening the characteristic hematological parameters in SLE patients

	B	SE	Wald	*P*‐value	Exp(B)	95% CI for EXP(B)	
						Lower	Upper
Gender	−1.265	1.053	1.443	.230	0.282	0.036	2.222
Age	−0.017	0.029	0.110	.740	0.990	0.936	1.048
WBC	0.703	0.470	2.242	.134	2.020	0.805	5.017
NEU	−1.529	0.573	7.108	.008*	0.217	0.070	0.667
LYM	−0.712	0.694	1.054	.305	0.490	0.126	1.911
NLR	1.393	0.417	11.141	.001*	4.028	1.777	9.128
RBC	−3.187	0.614	26.937	.000*	0.041	0.012	0138
HGB	−0.004	0.019	0.037	.848	0.996	0.960	1.034
HCT	0.000	0.010	0.000	.987	1.000	0.980	1.020
MCV	0.009	0.021	0.195	.659	1.009	0.969	1.051
RDW	4.080	3.155	20.203	.000*	2.008	1.482	2.712
PLT	−0.030	0.004	44.234	.000*	0.971	0.962	0.979
PLR	0.021	0.003	41.809	.000*	1.021	1.015	1.028
PCT	1.550	4.064	0.146	.703	4.714	0.022	1.357

Abbreviations: HCT, hematocrit; HGB, hemoglobin; LYM, lymphocyte; MCV, mean corpuscular volume; MPV, mean platelet volume; NEU, neutrophil; NLR, neutrophils‐to‐lymphocytes ratio; PCT, plateletcrit; PDW, platelet distribution width; PLR, platelet‐to‐lymphocyte ratio; PLT, platelet; RBC, red blood cell; RDW, red blood cell distribution width; WBC, white blood cell.

* *P* < .05 was regarded as statistically significant.

**Table 3:** Correlation of NLR, RDW, and PLR with immune, inflammatory, and metabolic indexes in SLE patients

	NLR		RDW		PLR	
	*r*	*P*‐value	*r*	*P*‐value	*r*	*P*‐value
IgG	.048	.484	−.033	.628	.263	.000*
IgM	.191	.092	.094	.247	.112	.133
IgA	.108	.246	.118	.235	.076	.338
C3	−.265	.000*	−.207	.002*	−.033	.629
C4	−.033	.630	−.196	.004*	−.015	.824
ESR	.635	.000*	.124	.100	.097	.242
Hs‐CRP	.469	.000*	.259	.017*	.193	.012*
CAR	.244	.003*	.248	.001*	.077	.318
TG	.383	0.000*	.326	.000*	.011	.884
TC	−.062	.420	−.015	.840	−.083	0.275
HDL‐C	−.033	.664	−.071	.354	−.275	.000*
LDL‐C	−.237	.002*	−.012	.877	−.051	.509
TP	.039	.581	−.257	.001*	.108	.127
PA	−.132	.061	−.131	.064	−.144	.041*
ALB	−.364	.000*	–.192	.006*	−0.028	.691
UA	.374	.000*	.064	.363	−.187	.008*
SLEDAI	.346	.000*	.279	.000*	.312	.000*

Abbreviations: ALB, albumin; C3, complement 3; C4, complement 4; CAR, c‐reactive protein level to albumin level ratio; ESR, erythrocyte sedimentation rate; HDL‐C, high‐density lipoprotein cholesterol; hs‐CRP, hypersensitive c‐reactive protein; IgA, immunoglobulin A; IgG, immunoglobulin G; IgM, immunoglobulin M; LDL‐C, low‐density lipoprotein cholesterol; PA, prealbumin; SLEDAI, Systemic Lupus Erythematosus Disease Activity Index; TC, total cholesterol; TG, triglyceride; TP, total protein; UA, uric acid.

* *P* < .05 was regarded as statistically significant.

**Table 4:** Association rules between NLR, RDW, and PLR and immune, inflammatory, and metabolic indexes

Former item	Consequent item	Support (%)	Confidence (%)
C3 + TG	NLR	23.163	58.619
hs‐CRP		31.441	62.731
C3		25.033	67.250
TG		28.712	63.583
ALB		22.622	61.773
UA		30.532	60.241
C3 + ALB	RDW	34.228	53.947
C4		28.053	63.162
hs‐CRP		32.339	55.545
TG		30.794	60.721
IgG	PLR	24.553	65.536
hs‐CRP		32.377	54.602
HDL‐C		28.464	61.443
UA		29.173	57.361

Abbreviations: NLR, neutrophils‐to‐lymphocytes ratio; PLR, platelet‐to‐lymphocyte ratio; RDW, red blood cell distribution width.
