# Supplementary material for: Hematological factors associated with immunity, inflammation, and metabolism in patients with systemic lupus erythematosus: Data from a Zhuang cohort in Southwest China
Source: J Clin Lab Anal. 2020 Jan 24;34(6):e23211. doi: 10.1002/jcla.23211 (PMC7307337; doi:10.1002/jcla.23211)
Supplement: Supplementary file 1 [file JCLA-34-e23211-s001.doc]

*Supplementary Table S1. Demographic, clinical* *fetures and laboratory findings of the study population.*

|  | SLE patients (n=195) | Healthy control (n=183) | P-value |
| --- | --- | --- | --- |
| Age (years) | 34(27,43) | 36(30,42) | 0.516 |
| Gender (Male/Female) | 17/178 | 18/165 | 0.708 |
| Ethnicity | China, Zhuang | China, Zhuang |  |
| SLEDAI scores | 8.25±6.16 |  |  |
| WBC (×10^9^/L) | 4.32(3.07,6.66) | 6.24(5.25,7.33) | <0.001 |
| NEU(×10^9^/L) | 2.69(1.84,4.42) | 3.55(2.86,4.45) | <0.001 |
| LYM (×10^9^/L) | 1.46(0.96,1.82) | 2.06(1.80,2.48) | <0.001 |
| RBC (×10^12^/L) | 3.85(3.34,4.47) | 4.63(4.35,4.87) | <0.001 |
| HGB(g/L) | 112(96,125) | 133(126,140) | <0.001 |
| HCT(%) | 30.8(27,36.6) | 39.4(37.5,41.0) | <0.001 |
| MCV(fL) | 83.6(78.2,88.5) | 86.6(81.6,88.8) | 0.007 |
| RDW(%) | 14.3(13.4,16.3) | 12.9(12.4,13.6) | <0.001 |
| PLT(×10^9^/L) | 192(145,255) | 295(254,338) | <0.001 |
| MPV(fL) | 9.9(9.3,10.63) | 9.9(9.3,10.5) | 0.755 |
| PDW(%) | 11.1(9.8,12.3) | 11.1(10.1,12.4) | 0.325 |
| PCT(%) | 0.2(0.2,0.3) | 0.29(026,0.33) | <0.001 |
| NLR | 2.70(1.61,4.74) | 1.67(1.31,2.01) | <0.001 |
| PLR | 173.5(102.5,252.3) | 135.3(112.1,163.1) | <0.001 |
| IgG(g/L) | 17.33±6.06 | 10.96±4.24 | <0.001 |
| C3(g/L) | 0.53(0.26,0.74) | 1.09(0.88,1.29) | <0.001 |
| C4(g/L) | 0.08(0.05,0.14) | 0.21(0.13,0.26) | <0.001 |
| hs-CRP (mg/L) | 2.85(0.86,15.22) | 0.72(0.33,4.13) | <0.001 |
| ESR (mm/H) | 39(18,73) | 14(7.5,22) | <0.001 |
| CAR | 0.075(0.022,0.479) | 0.002(0.002,0.021) | <0.001 |
| TP(g/L) | 63.77±11.74 | 70.43±4.04 | <0.001 |
| PA(mg/L) | 202±87.11 | 281±50.88 | <0.001 |
| ALB(g/L) | 34.75(28.35,40.92) | 45.75(43.90,47.45) | <0.001 |
| TC(mmol/L) | 5.01±1.06 | 4.62±1.44 | 0.003 |
| TG(mmol/L) | 1.41(0.98,2.20) | 1.16(0.88,1.54) | <0.001 |
| HDL-C(mmol/L) | 1.30±0.54 | 1.61±0.35 | <0.001 |
| LDL-C(mmol/L) | 2.57±1.07 | 2.63±0.70 | 0.521 |
| UA(umol/L) | 320(253.25,397.25) | 290(253.75,334.50) | 0.004 |

Abbreviations: SLEDAI: Systemic Lupus Erythematosus Disease Activity Index; WBC: [white](../../../../C:/Users/lenovo/AppData/Local/youdao/dict/Application/8.5.1.0/resultui/html/index.html#/javascript:;)  [blood](../../../../C:/Users/lenovo/AppData/Local/youdao/dict/Application/8.5.1.0/resultui/html/index.html#/javascript:;)  [cell](../../../../C:/Users/lenovo/AppData/Local/youdao/dict/Application/8.5.1.0/resultui/html/index.html#/javascript:;); NEU: neutrophil; LYM: lymphocyte; RBC: red [blood](../../../../C:/Users/lenovo/AppData/Local/youdao/dict/Application/8.5.1.0/resultui/html/index.html#/javascript:;) [cell](../../../../C:/Users/lenovo/AppData/Local/youdao/dict/Application/8.5.1.0/resultui/html/index.html#/javascript:;); HGB: [hemoglobin](../../../../C:/Users/lenovo/AppData/Local/youdao/dict/Application/8.5.1.0/resultui/html/index.html#/javascript:;); HCT: hematocrit; MCV: mean corpuscular volume; RDW: red blood cell distribution width; PLT: platelet; MPV: mean platelet volume; PDW: platelet distribution width; PCT: plateletocrit; NLR: neutrophils-to-lymphocytes ratio; PLR: platelet-to-lym-phocyte ratio; IgG: immunoglobulin; C3: complement 3; C4: complement 4; hs-CRP: hypersensitive c-reactive protein; ESR: erythrocyte sedimentation rate; CAR: c-reactive protein level-to-albumin level ratio; TP: total protain; PA: [prealbumin](../../../../C:/Users/lenovo/AppData/Local/youdao/dict/Application/8.5.1.0/resultui/html/index.html#/javascript:;); ALB: [albumin](../../../../C:/Users/lenovo/AppData/Local/youdao/dict/Application/8.5.1.0/resultui/html/index.html#/javascript:;); TC: total cholesterol; TG: triglyceride; HDL-C: high density lipoprotein cholesterol; LDL-C: low density lipoprotein cholesterol; UA: uric acid.
